# Supplementary material for: Epidemiological and Immunological Features of Obesity and SARS-CoV-2
Source: Viruses. 2021 Nov 6;13(11):2235. doi: 10.3390/v13112235 (PMC8624148; doi:10.3390/v13112235)
Supplement: Supplementary file 1 [file viruses-13-02235-s001.zip › viruses-1431531-supplementary.pdf]

**Table S1:** Characteristics and serostatus of South Texas site with high seropositive rate (22·5%) (N=712)

| Covariate[1] | All participants (n=712) | Seropositive participants (n=160) | | OR (95% CI) | P-Value[2] |
|---|---|---|---|---|---|
| | N | N | % | | |
| **Age group** | | | | | |
| **18-29 y** | 345 | 86 | 24.9% | ref | |
| **30-39 y** | 181 | 35 | 19.3% | 0.72 (0.46 to 1.12) | 0.1488 |
| **40-49 y** | 128 | 28 | 21.9% | 0.84 (0.52 to 1.37) | 0.4907 |
| **50-59 y** | 45 | 10 | 22.2% | 0.86 (0.41 to 1.81) | 0.6921 |
| **60+ y** | 12 | 1 | 8.3% | 0.27 (0.03 to 2.15) | 0.2179 |
| **Body mass index** | | | | | |
| **<18.5** | 8 | 1 | 12.5% | 0.35 (0.04 to 2.91) | 0.3291 |
| **18.5-<25** | 144 | 42 | 29.2% | ref | |
| **25-<30** | 230 | 53 | 23.0% | 0.73 (0.45 to 1.17) | 0.1864 |
| **30-<35** | 162 | 34 | 21.0% | 0.65 (0.38 to 1.09) | 0.0995 |
| **35-<40** | 63 | 14 | 22.2% | 0.69 (0.35 to 1.39) | 0.3022 |
| **≥40** | 22 | 1 | 4.5% | 0.12 (0.02 to 0.89) | 0.0380* |
| **Ethnicity** | | | | | |
| **Not Hispanic/Not Latinx** | 93 | 16 | 17.2% | ref | |
| **Hispanic/Latinx** | 493 | 117 | 23.7% | 1.50 (0.84 to 2.67) | 0.1702 |
| **Race** | | | | | |
| **White** | 375 | 82 | 21.9% | ref | |
| **American Indian/Alaska Native** | 9 | 3 | 33.3% | 1.79 (0.44 to 7.30) | 0.4190 |
| **Asian** | 9 | 3 | 33.3% | 1.79 (0.44 to 7.30) | 0.4190 |
| **Black** | 7 | 1 | 14.3% | 0.60 (0.07 to 5.02) | 0.6336 |
| **Native Hawaiian/Pacific Islander** | 3 | 0 | 0.0% | 0.00 (0.00 to Inf) | 0.9792 |
| **More than one race** | 26 | 4 | 15.4% | 0.65 (0.22 to 1.94) | 0.4393 |
| **Sex** | | | | | |
| **Female** | 44 | 10 | 22.7% | ref | |
| **Male** | 634 | 144 | 22.7% | 1.00 (0.48 to 2.07) | 0.9982 |
| **Children ≤ 18 y in household** | | | | | |
| **No** | 407 | 102 | 25.1% | ref | |
| **Yes** | 273 | 54 | 19.8% | 0.74 (0.51 to 1.07) | 0.1091 |
| **No. in household** | | | | | |
| **1** | 98 | 22 | 22.4% | ref | |
| **2-4** | 393 | 97 | 24.7% | 1.13 (0.67 to 1.92) | 0.6446 |
| **>4** | 173 | 34 | 19.7% | 0.84 (0.46 to 1.55) | 0.5852 |
| **Comorbidity[3]** | | | | | |
| **Asthma** | 27 | 4 | 14.8% | 0.59 (0.20 to 1.73) | 0.3364 |
| **Hypertension** | 47 | 11 | 23.4% | 1.06 (0.53 to 2.13) | 0.8741 |
| **Diabetes mellitus** | 15 | 3 | 20.0% | 0.86 (0.24 to 3.09) | 0.8168 |
| **Other lung disease** | 2 | 1 | 50.0% | 3.47 (0.22 to 55.71) | 0.3805 |
| **Other chronic medical** | 9 | 2 | 22.2% | 0.99 (0.20 to 4.79) | 0.9856 |
| **Smoking history** | | | | | |
| **Never** | 546 | 124 | 22.7% | ref | |
| **Prior** | 58 | 15 | 25.9% | 1.18 (0.64 to 2.20) | 0.5933 |
| **Current** | 80 | 17 | 21.3% | 0.92 (0.52 to 1.62) | 0.7641 |

[1] Not reported data: age group (n=1), BMI (83), ethnicity (126), race (283), sex (34), children in HH (31), No. in HH (47), comorbidities (28).

[2] P-values unadjusted for multiple hypothesis testing. * P<0.05

[3] Other comorbidities with no seropositive individuals (n= number reporting comorbidity): cononary heart disease (n=3), chronic kidney disease (2), stroke (2), COPD (0), Heart failure (0), cancer receiving treatment (1), cancer not receiving treatment (3), other heart disease (0), other immunocompromised (0).

**Table S2:** Symptoms reported by healthy/normal versus overweight but not obese seropositive individuals (n=178)

| Clinical feature | Normal weight (18.5-<25) (n=89) | | Overweight (25-<30) (n=89) | | P-value[1] |
|---|---|---|---|---|---|
| | N | % | N | % | |
| Fever | 7 | 7.9% | 6 | 6.7% | 0.76 |
| Chills | 9 | 10.1% | 11 | 12.4% | 0.63 |
| Cough | 16 | 18.0% | 14 | 15.7% | 0.68 |
| Loss of smell | 9 | 10.1% | 14 | 15.7% | 0.27 |
| Loss of taste | 8 | 9.0% | 11 | 12.4% | 0.46 |
| Nausea and/or vomiting | 5 | 5.6% | 5 | 5.6% | 1.00 |
| Diarrhea | 7 | 7.9% | 11 | 12.4% | 0.32 |
| Congestion | 18 | 20.2% | 17 | 19.1% | 0.85 |
| Sore throat | 11 | 12.4% | 14 | 15.7% | 0.54 |
| Myalgias | 13 | 14.6% | 11 | 12.4% | 0.67 |
| Increased fatigue | 23 | 25.8% | 14 | 15.7% | 0.10 |
| **Aggregate symptom measures[2]** | | | | | |
| Any symptom | 36 | 40.4% | 35 | 39.3% | 0.88 |
| ≥3 symptoms | 19 | 21.3% | 20 | 22.5% | 0.85 |
| ≥6 symptoms | 11 | 12.4% | 8 | 9.0% | 0.46 |
| Any primary symptom | 20 | 22.5% | 22 | 24.7% | 0.73 |
| ≥3 primary symptoms | 10 | 11.2% | 10 | 11.2% | 1.00 |
| Symptoms count (mean) | 126 | 1.42 | 128 | 1.44 | 0.96 |
| Primary symptom count (mea | 49 | 0.55 | 56 | 0.63 | 0.68 |

[1] Chi-squared test of proportions and ANOVA of means

[2] Primary symptoms: Fever, chills, cough, loss of smell and loss of taste

**Table S3:** Results of univariate differences (Mann-Whitney U test) in immune features by obese (n=25) versus non-obese (n=52) status

| Feature | p-value |
|---|---|
| FcgR3b-RBD | 2·84E-01 |
| FcgR3b-S | 2·30E-01 |
| FcgR3b-N | 1·70E-01 |
| FcgR3b-NTD | 2·47E-01 |
| IgA1-N | 3·14E-01 |
| IgA1-S | 1·41E-01 |
| IgA1-NTD | 3·32E-01 |
| IgA1-RBD | 2·38E-01 |
| IgG1-N | 1·33E-01 |
| IgG1-NTD | 1·09E-01 |
| IgG1-S | 2·23E-01 |
| IgG1-RBD | 3·20E-01 |
| IgG3-N | 3·51E-01 |
| IgG3-S | 4·38E-01 |
| IgG3-NTD | 4·24E-01 |
| IgG3-RBD | 1·51E-01 |
| IgM-RBD | 2·23E-01 |
| IgM-S | 2·03E-01 |
| IgM-NTD | 2·84E-01 |
| IgM-N | 1·45E-01 |
